# Analyzing Children's Weight Growth Variations and Associated Factors in Ethiopia, India, Peru, and Vietnam: Using Fractional Polynomial Mixed-Effects Model

**DOI:** 10.4314/ejhs.v34i1.4

**Published:** 2024-01

**Authors:** Alemayehu Siffir Argawu, B Muniswamy, B Punyavathi

**Affiliations:** 1 Department of Statistics, College of Science and Technology, Andhra University, India

**Keywords:** Children, Weight Growth, Growth Rate, Linear Mixed-Effects Mode

## Abstract

**Background:**

Children's growth is increasingly considered a key mediator of later life outcomes. When examining weight growth, the correlation between repeated observations on the same subject must be regarded as well-modelled. This study aimed to analyze children's weight growth variations and associated factors in Ethiopia, India, Peru, and Vietnam using a fractional polynomial mixed-effects model.

**Methods:**

This study used longitudinal data from the Young Lives Cohort Study conducted from 2002 to 2016 in Ethiopia, India, Peru, and Vietnam. The study included 7,140 children of 1 to 15 years old A fractional polynomial mixed-effects model was used to analyze the data.

**Results:**

Ethiopian, Peruvian, and Vietnamese children had significantly higher average body weights than children in India (1.426, P<0.001; 1.992, P<0.001; 1.334, P<0.001, respectively). Girl children's average body weight was significantly 0.15 times less than that of boys (−0.148; P=0.027). The average weight of rural children was significantly 0.671 times less than that of urban children (0.671, P<0.001). Children from Peru and Vietnam had higher rates of weight change than those from India. However, the rate of weight change was lower in Ethiopian children than in Indian children. Children from urban areas had a significantly higher rate of weight gain than those from rural areas.

**Conclusion:**

Country, sex, residence, parental education, household size, wealth, good drinking water, and reliable power affected children's longitudinal weight growth. Therefore, WHO and the nation's health ministry should monitor children's weight growth status and these associated factors to plan future action.

## Introduction

Children's physical growth rate is the most accurate indication of a child's physical health worldwide (1, 2). Children's growth is increasingly considered a key mediator of later life outcomes (3, 4). Modelling children's growth variables is necessary to study weight growth (5).

Traditional general regression uses a single equation or growth curve for the entire sample and ignores individual growth differences. Traditional regression analyses hierarchical data, which may produce misleading standard errors and p-values (6, 7). Mixed-effects regression estimates individual and sample average curves using individual growth features (8). When studying repeated outcomes like children's weight or height, a correlation between repeated observations of the same person should be examined (9). It can be done with a mixed-effects model. Mixed-effects models are often used to characterize trajectories because they cluster repeated measures (Level 1) within individuals for repeated analysis (Level 2). One method is to represent weight as a function of age. This function can be linear or polynomial. Children's growth is often assessed by weight, height, and head circumference. Evaluating children's development is a typical way to discover growth failure and its association with adverse circumstances.

This longitudinal data has been used to study children's features in four low- and middle-income countries: Ethiopia, India, Peru, and Vietnam. Kumar, Kumar (10) looked at heterogeneity effects on children's height and weight gain at 14–15 years, Humphries, Dearden (11) studied household food expenditure and child anthropometry at 5, 8, and 12 years, Aurino, Schott (12) observed nutritional status and adolescent learning from 1 to 15 years. Tran, Holton (13) investigated children's physical growth as an indicator of early childhood development. Wake, Zewotir (14), (15) studied children's nonlinear height and height growth features. However, none of these studies have examined children's weight growth variation and its associated factors in the four low- and middle-income countries. Thus, this study aimed to analyze children's weight growth variations in Ethiopia, India, Peru, and Vietnam using a fractional polynomial mixed-effects model. In addition, this study identified associated factors on children's weight gain.

## Materials and Methods

**Data source and study participants**: Young Lives, a 15-year longitudinal study on child poverty in four low- and middle-income countries (Ethiopia, India, Peru, and Vietnam), collected longitudinal data (16, 17). Five weight measurements were taken from 1–15-year-olds in this study. Seven thousand one hundred forty children from the four nations provided 35,700 observations (n = 5 per child).

**Variable Types**: Anthropometric measurements in children are based on WHO criteria (18, 19). The response variable was the child's scale-measured weight (kg). Independent variables included age, sex, household size, wealth index, country, residential area, father's and mother's ages, educational levels, safe drinking water, sanitation, and quality electricity.

**Statistical analysis**: When modeling longitudinal data, mixed-effect models are frequently used to test hypotheses about intra-individual changes and inter-individual variability in intra-individual changes (20, 21). Therefore, the linear mixed-effect model (22, 23) can be expressed as follows:


$$y_{i}=X_{i}\beta +Z_{i}b_{i}+\varepsilon_{i}.\;\;\;\;\;\;\;(1)$$

where y_i_ is the (n_i_ × 1 vector of continuous outcomes for the i^th^ individual (child), and it is assumed that y_ij_ represents the result score for the i^th^ (1,2,3, …, n = 7,140) chlid at the j^th^ (1, 2, 3, 4, 5) measurement event. X_i_ is an (n_i_ × p) covanate matrix related to the fixed effects β, where β∈ R^p×1^. Z_i_ is the (n_i_ × q) design matrix related to random effects b_i_, where b_i_∈ R^q×1^, and ε_i_ is the (n_i_ × 1) within-individual error vector.

It is linear if the trajectory change is assumed linear and the shape's form is not in question. Nevertheless, a common trajectory shape was selected from various possibilities when the change was nonlinear. For instance, fractional polynomials that enable nonlinear trajectories were presented by Royston and Altaian (24). The following definition applies to an m-order fractional polynomial with power p_j_:


$$\phi_{m}(t;\beta ,p)=\beta_{0}+\sum_{j=1}^{m}\beta_{j}t_{\text{ij}}^{p_{j}}j=(1,2,\ldots ,m).\;\;\;\;\;\;\;(2)$$

where p = (p_1_, …, p_m_) is a real-valued vector of powers, m is a positive integer representing the order of the polynomial, and β = (β_0_, …, β_m_) is the coefficient. The power terms were selected from a predefined set of integers and non-integers for growth-fitting applications, p_j_ = (− 2, − 1, − 0.5, 0, 0.5, 1, 2, 3) Among all possible combinations of these powers, the best-fitting model is evaluated based on its deviance. AIC, and BIC information criteria, where the smallest is better (24). A fractional polynomial mixed-effects model at the individual level is expressed as follows, using Equations 1 and 2:


$$y_{ij}=\beta_{0}+\sum_{j=1}^{m}\beta_{j}t_{ij}{\;}^{p}+b_{i0}+\sum_{j=1}^{m}b_{ij}t_{ij}{\;}^{p_{j}}+e_{ij}.$$

**Ethics**: Because this study used a secondary data from Young Lives Study that is publicly available in the link below so additional ethical approval was not required. The Young Lives data are accessible to anyone who visits the website http://www.younglives.org.uk/.

## Results

**Exploratory data analysis**: Each child's growth weight was measured five times from 1 to 15 years old in 35,700 observations. There were 9,310 (26.1%), 8,325 (23.3%), 8,805 (24.7%), and 9,260 (25.9%) observations from India, Ethiopia, Peru, and Vietnam, respectively, presented by observations in (Table 1).

**Table 1:** Frequency distribution of the categorical variables by the number of observations

Variables and Categories	Observations (%)
**Country**	
India	9,310 (26.1)
Ethiopia	8,325 (23.3)
Peru	8,805 (24.7)
Vietnam	9,260 (25.9)
**Sex of child**	
Girl	17,060 (47.8)
Boy	18,640 (52.2)
**Residence area**	
Rural	22,129 (62)
Urban	13,571 (38)
**Father's education level**	
Uneducated	6,179 (17.3)
Adult literacy and religious	894 (2.5)
Grade 1-4	4,974 (13.9)
Grade 5-8	12,382 (34.7)
Grade 9-12	8,321 (23.3)
Diploma and above	2,950 (8.3)
**Mother's education level**	
Uneducated	10,264 (28.7)
Adult literacy and religious	853 (2.4)
Grade 1-4	6,324 (17.7)
Grade 5-8	8,806 (24.7)
Grade 9-12	7,241 (20.3)
Diploma and above	2,212 (6.2)
**Access to safe drinking water**	
No	13,811 (38.7)
Yes	21,889 (61.3)
**Access of sanitation**	
No	13,002 (36.4)
Yes	22,698 (63.6)
**Access to quality electricity**	
No	6,633 (18.6)
Yes	29,067 (81.4)

Table 2 shows the means, variance-covariance, and correlations of weight measurements taken on the same child at 1, 5, 8, 12, and 15 years of the mean age in each round (1 to 5). Average weight increases at different rates with age. It implies that linear models cannot predict weight gain. Weight measurements on the same child at different times and near each other were more likely to be correlated than those taken farther apart. Therefore, the analysis must include related measurements (Table 2).

**Table 2:** Correlation and covariance matrix for weight by age

Weight	Age 1	Age 5	Age 8	Age 12	Age 15
Age 1	1.92	2.46	3.62	6.21	7.04
Age 5	0.66	7.29	10.18	17.44	18.48
Age 8	0.60	0.86	19.14	31.96	33.73
Age 12	0.52	0.74	0.84	75.71	73.83
Age 15	0.50	0.68	0.76	0.84	102.56
Mean	8.35	16.27	21.69	34.31	46.61

Variances on the diagonal, covariances above the diagonal, and correlations below the diagonal

**Figure 1:**
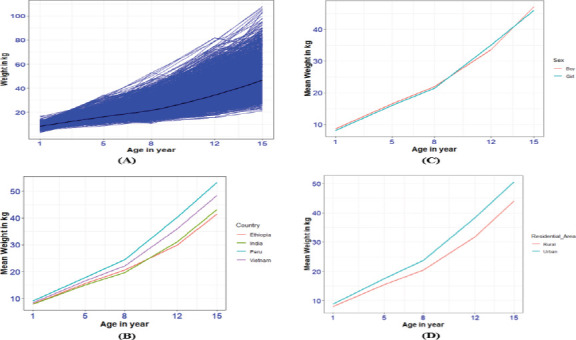
Children's weight growth (A) Overall Children's weight growth, (B) Mean weight growth by country, (C) Mean weight growth by child's sex, and (D) Mean weight growth by residential area

The overall profde and smooth loess curves for children's weight growth are shown (Figure 1-A). A rapid increase in weight characterizes children in adolescence. The group profde plots of mean weight increase across countries show a variation in mean weight gain between countries and that age has a stronger relationship with mean weight gain. Peruvian children had higher mean weight gain than children in other countries. Indian and Ethiopian children had lower mean weight gain earlier and later, respectively (Figure 1-B). The mean weight gain of boys was somewhat higher than that of girls (Figure 1-C). Children in urban areas grew faster on average than those in rural areas (Figure 1-D). These figures show a nonlinear functional relationship between age and weight.

**Results from the fitted fractional polynomial mixed-effects model**: Children's weight increase was analyzed by age or time. The time variable was described by power terms p and q = −2, −1, −0.5, 0. 0.5, 1, 2, and 3. After comparison, the model with p = −2 and q = 2 had the lowest deviation. Thus, the fractional polynomial functions that best suited the children's weight data were p = −2 and q = 2. It is how aging influences weight growth. The age effect model was upgraded with fixed and random effects. Equation (3) can be rearranged as follows:


$$\beta_{0}+b_{0i}+(\beta_{1}+b_{1i})t^{-2}+(\beta_{2}+b_{2i})t^{2}+\varepsilon .$$

The population parameters β_0_, β_1_, and β_2_ reflect the intercept, inverse square of time (t^−2^), and square of time (t^2^), effects, respectively. The random effects b_0i_, b_1i_, and b_2i_ affect children's recurrent weight gain over time. Individuals' influence on longitudinal growth statistics is more likely positive or negative (25). Random factors in the model explain weight increase variation between individuals. Thus, the model must include random effects to account for individual differences. Next, we utilized likelihood ratio test statistics to evaluate the models with intercept, square age, and inverse square age random effects. Finally, likelihood ratio test statistics indicated that an intercept-slope model was optimum. We choose the optimum error term covariance structure using likelihood-based information criteria AIC, BIC, and deviation; the smaller, the better. We found that the heterogeneous unstructured covariance fit the model best.

**Estimated coefficients from the fixed-effects model**: The effects of fixed covariates, such as a fractional polynomial function of age (square age and inverse square age), country, child's sex, child's residence area, household size, household wealth index, father's age, mother's age, father's education level, mother's education level, access to safe drinking water, access to sanitation, access to good quality electricity, and the interaction effect of age functions with sex, residence area, and country were included in the model and examined. The fractional polynomial fixed effects model results are presented (Table 3).

**Table 3:** Results of the fixed-effects of the fractional polynomial mixed-effects model's parameters estimates

Effect	Estimate	Std Error	t-value	P-value
**Intercept**	11.394	0.176	64.593	<0.001*
**Age ^−2^**	-3.780	0.117	-32.303	<0.001*
**Age^2^**	0.147	0.001	126.455	<0.001*
**Country (Ref: India)**				
Ethiopia	1.426	0.105	13.541	<0.001*
Peru	1.992	0.107	18.537	<0.001*
Vietnam	1.334	0.107	12.513	<0.001*
**Child's sex (Girl)**	-0.148	0.067	-2.212	0.027*
**Residential Area (Rural)**	-0.671	0.081	-8.303	<0.001*
**Household size**	-0.039	0.009	-4.204	<0.001*
**Wealth index**	1.302	0.167	7.793	<0.001*
**Father's Age**	-0.006	0.004	-1.439	0.150
**Mother's Age**	0.008	0.005	1.590	0.112
**Father's education level (Ref: Uneducated)**				
Adult literacy and religious	-0.840	0.220	-3.740	0.691
Grade 1-4	-0.272	0.074	-3.681	<0.001*
Grade 5-8	-0.035	0.066	-0.527	0.598
Grade 9-12	0.123	0.076	1.617	0.106
Diploma or above	0.255	0.113	2.255	0.024*
**Mother's education level (Ref: Uneducated)**				
Adult literacy and religious	0.185	0.189	0.982	0.326
Grade 1-4	-0.092	0.069	-1.332	0.183
Grade 5-8	0.070	0.068	1.022	0.307
Grade 9-12	0.440	0.080	5.497	<0.001*
Diploma or above	0.929	0.127	7.291	<0.001*
**Access to safe drinking water (Yes)**	0.154	0.049	3.168	0.002*
**Access to sanitation (Yes)**	-0.027	0.046	-0.575	0.566
**Access to quality electricity (Yes)**	-0.342	0.059	-5.818	<0.001*

The intercept factor's mean indicates that the model-implied child's weight at age one was 11.394 kg. Body weight changed significantly over the age of the inverse square (age^−2^:−3.78; P<0.001) and square age (age^−2^:0.147; P<0.001) for the age effect. A positive impact of age squared indicates that the effect on weight gain becomes more substantial as children age. Compared to children in India, Ethiopia, Peru, and Vietnam had significantly higher average body weights (Ethiopia: 1.426, P<0.001; Peru: 1.992, P<0.001; and Vietnam: 1.334, P<0.001). The average body weight of girls was significantly 0.15 times lower than that of boys, indicating a considerable sex disparity in average body weight (girls: -0.148; p = 0.027). The average body weight of rural children was 0.671 times lower (rural: -0.671; P<0.001) than urban children. It indicates that the residential area of the children significantly affects weight gain.

The educational levels of fathers and mothers are also associated with their children's weight. The positive average differences in weight growth between children of most educated parents and children of uneducated parents showed that the children of most educated parents (diploma or above:0.255; p = 0.024 for fathers, diploma or above:0.929; P<0.001; and grade 9–12:0.44; P<0.001 for mothers). Furthermore, the negative mean differences in weight growth between children of less-educated fathers (grades 1–4: - 0.54; P<0.001) had significantly lower mean weights than those of uneducated fathers. However, there was no significant difference in mean weight between children of uneducated parents and children of fathers (adult literacy and religion: -0.84; p = 0.691, grade 5–8: -0.035; p = 0.598, and grade 9–12:0.123; p = 0.106) and children of mothers (adult literacy and religion:0.185; p = 0.326, grade 1–4: -0.092; p = 0.183, and grade 5–8:0.07; p = 0.307).

The wealth index has a significant positive effect on child growth (1.302; P<0.001), while household size has a significant negative impact (household size: -0.039; P<0.001). Access to safe drinking water and good quality electricity significantly positively and negatively influenced children's weight growth (access to safe drinking water:0.154; p = 0.002, and access to quality electricity: -0.342; P<0.001), respectively. However, covariates such as paternal age. maternal age, and good sanitation access did not affect the child's weight growth.

Estimated parameters from the random-effects model: The estimated variances from the fitted fractional polynomial mixed-effects model are also presented. As a result, the residual variance (σ^2^) equals 5.581, and the variance of the random intercept ($\sigma_{b0}^{2}$) equals 3.131, representing the estimated variance in within-individual (child) variations. Thus, the intraclass correlation coefficient (ICC) σb02(σb02+σ2) was 0.36. It shows that compared to the natural variance within children, 36% of the variability in a child's weight can be attributed to changes across children. A large ICC indicates a strong correlation between observations within a child. The random slope estimated variances ($\sigma_{b1}^{2}$ and $\sigma_{b2}^{2}$) for the random covariates (inverse square age (time) and square age (time)) are 1.923 and 0.0013, respectively. The computed correlation coefficients are -0.98 and 0.25 between the random intercept and the two random slopes, respectively, and -0.07 between the two random slopes (Table 4).

**Table 4:** The result from the fitted fractional polynomial random effect model's parameters estimates

Groups	Name	Variance	SD	Correlation	
Individual	Intercept	3.131	1.769		
	Age^−2^	1.923	1.387	−0.98	
	Age^2^	0.0013	0.0355	0.25	−0.07
Residual		5.581	2.362		
Number of observations = 35,700, and groups (individuals) = 7,140					

**Rate of changes in body weight gain**: The average differences between the first and second weight measurements are 7.92 kg, 5.42 kg between the second and third measurements, 12.62 kg between the third and fourth measurements, and 12.3 kg between the fourth and fifth measurements. Although the increases in the means over time are not constant, they are more prominent in magnitude for later years than for earlier years (Table 2). Therefore, the curves for the weight increase may not be straight. Therefore, the square age and inverse square age functions are the best fits for analyzing the nonlinearity of the weight growth trajectories over time. The slope of the tangent line of the curve at age one, represented by the mean of the square inverse age factor, is -3.78. Accordingly, children's growth trajectories should have an average upbeat squared inverse growth section. The quadratic age factor's mean value is 0.147, which means that, on average, the curve becomes steeper as the child ages. These results demonstrate that children's weight increases along a developmental trajectory across time, with the degree of change increasing as the children get old. The interaction effects between these factors and the age functions are presented (Table 5).

**Table 5:** The weight gain rate of children by country, child's sex, and residential area

Effect	Estimate	SE	t-value	p-value
Age × Country (Ref: India)				
Age^−2^ × Ethiopia	-1.000	0.121	-8.237	<0.001*
Age^−2^ × Peru	-0.907	0.127	-7.167	<0.001*
Age^−2^ × Vietnam	-0.852	0.118	-7.250	<0.001*
Age^2^ × Ethiopia	-0.014	0.001	-10.632	<0.001*
Age^2^ × Peru	0.033	0.001	24.570	<0.001*
Age^2^ × Vietnam	0.020	0.001	15.568	<0.001*
Age × Sex				
Age^−2^ × Girl	-0.370	0.084	-4.343	<0.001*
Age^2^ × Girl	-0.002	0.001	-1.721	0.085
Age × Residential Area				
Age^−2^ × Rural	0.776	0.096	8.119	<0.001
Age^2^ × Rural	-0.010	0.001	-11.846	<0.001

Different weight increase rates were observed according to country, sex, and residential area. Regarding weight gain, children from four countries (Ethiopia, India, Peru, and Vietnam) showed considerable growth variation. Children from Peru and Vietnam had higher rates of change than children from India concerning weight increase. However, the rate of change was noticeably lower in Ethiopian children. When sex and inverse square age were considered, girls gained weight considerably slower than boys. However, when considering the interaction effects of sex and square age, there was no discernible difference in weight growth rate between boys and girls. Children from urban areas had a significantly higher rate of weight gain than those from rural areas. The rates of weight gain accelerated more in the later years than in the earlier years.

**India**: ŷ_ij_ = Constant_IND_ − 3.78 ∗ (Age)^−2^ + 0.147 ∗ (Age)^2^.

**Ethiopia**: ŷ_ij_ = Constant_ETH_ − 4.773 ∗ (Age)^−2^ +0.1333 ∗ (Age)^2^.

**Peru**: ŷ_ij_ = Constant_FER_ − 4.687 ∗ (Age)^−2^ +0.1803 ∗ (Age)^2^.

**Vietnam**: ŷ_ij_ = Constant_VIT_ − 4.642 ∗ (Age)^−2^ +0.167 ∗ (Age)^2^.

Calculating the instantaneous rate of change for each country using the marginal equations for that country yields the following possible form for that country's rate:

**India**: $\frac{d{\hat{y}}_{ij}}{d(\text{time})}=7.56\;*\;(\text{Age}{)}^{-3}+0.294\;*\;(\text{Age})$.

**Ethiopia**: $\frac{d{\hat{y}}_{ij}}{d(\text{time})}=9.546\;*\;(\text{Age}{)}^{-3}+0.2666\;*\;(\text{Age})$.

**Peru**: $\frac{d{\hat{y}}_{ij}}{d(\text{time})}=9.374\;*\;(\text{Age}{)}^{-3}+0.3606\;*\;(\text{Age})$.

**Vietnam**: $\frac{d{\hat{y}}_{ij}}{d(\text{time})}=9.284\;*\;(\text{Age}{)}^{-3}+0.334\;*\;(\text{Age})$.

**Figure 2:**
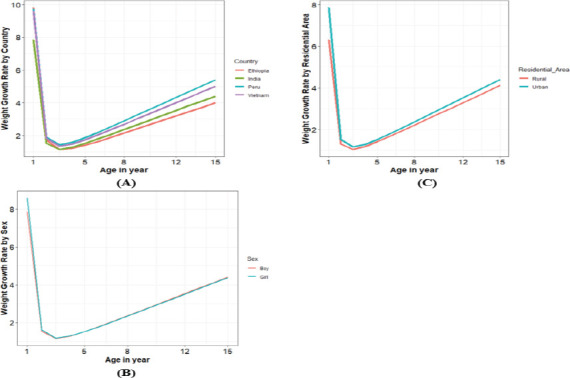
Plots of children's weight growth rate by (A) country, (B) child's sex, and (C) residential area

Likewise, we can find functions for children's weight growth rates according to sex and residential area categories. Moreover, plots of children's weight growth rates by country, sex. and residential area are presented in Figure (Figure 2-A, B, and C).

## Discussion

This study employed a longitudinal analysis to analyze how children's weight increases over time and how different factors affect their growth. This study found a nonlinear functional link between children's age and weight increase. Age transformation is required to reflect this nonlinear trend in weight growth. The best-fitting age transformation was discovered using a fractional polynomial technique, and the inverse square age (Age^−2^) and square age (Age^2^) were selected as the best-fitting functions for the weight increase.

A higher weight growth rate was observed in Peru after early infancy (age 3). In contrast, it is low in Ethiopia after early infancy (age three years) and in India during the initial years of life (ages 1–3 years). It indicates that Ethiopia and India have inadequate public health and governance. Poor governance hinders long-lasting the ability of health outcomes (26). Studies have shown that African children's growth patterns fall below those recommended by WHO (27, 28). Additionally, socioeconomic disparities between countries may cause children's weight growth discrepancies in low- and middle-income countries (29, 30).

Child sex greatly affected weight gain, with boys significantly heavier than girls. According to Adenaike, Peters (31) and Demerath, Choh (32). this may result from genetic differences. Additionally, a study from Ethiopia suggests that female children were more likely than male children to be born with low birth weights (33). This result is consistent with earlier studies that discovered that boys were heavier than girls (34). Urban versus rural residency substantially impacted children's weight gain trends, indicating that urban children were significantly more severe than rural areas, validating earlier research findings (35, 36). Children's growth rates in urban and rural locations may differ because of socio-environmental variables and nutritional conditions (21).

Children of more educated mothers and fathers were heavier than their counterparts, consistent with the previous studies' findings that children of educated parents are more severe than their counterparts (35, 37). Mothers' and fathers' education significantly impacted children's weight growth. According to Hosseini, Maracy (34), babies born to mothers with lower levels of education are considerably smaller at birth than babies delivered to mothers with higher levels of education. This is because there is a favorable correlation between parental education, whether from the mother or father (38).

The study found that neither the mother's nor the father's age significantly affected the children's weight growth trajectory. This finding aligns with earlier research (35, 39) that parents' age had no significant impact on the child's growth trajectory. Moreover, the wealth index was positively correlated with children's growth trend, whereas household size was negatively correlated with children's weight increase, consistent with other studies (30, 35). It supports earlier research findings (40) that home structure is associated with children's concurrent stunting and being overweight. In addition, home access to clean water and reliable electricity had significant favorable and unfavorable impacts on the trend in children's weight gain, respectively. In contrast, there was no significant association between household sanitation access and increased child weight.

Our study has some limitations, mostly related to the small number of variables (like birth weight, body mass index (BMI), a child's nutritional status, and health-care consumption) that were not taken into account. We, therefore, recommended that the Young Lives Study incorporate these variables (listed above) in their cohort study and that the model used in this study be extended to encompass a variety of time-invariant and time-varying variables in future research.

In conclusion, this study's results contribute to understanding children's weight growth variations and their associated factors in four low-income and middle-income countries. The longitudinal weight growth of the children was significantly influenced by country, sex, residence, parental education, household size, wealth, access to safe drinking water, and access to reliable electricity. However, the education levels of the mother and father and access to sanitation were not associated with the trajectory of weight gain over time. There was a variation in the children's weight growth rates among the four LMICs. Furthermore, Peru has a higher child weight growth rate during early infancy. In contrast, it is low in Ethiopia after early infancy and in India during the initial years of life. The findings of this study can be used to identify disparities in children's weight growth. Therefore, interested organizations like WHO and the nation's Ministry of Health should pay close attention to children's weight growth status and these related factors to plan for further action.
